# The school education, ritual customs, and reciprocity associated with self-regulating hand hygiene practices during COVID-19 in Japan

**DOI:** 10.1186/s12889-022-14012-z

**Published:** 2022-09-02

**Authors:** Sun Youn Lee, Shusaku Sasaki, Hirofumi Kurokawa, Fumio Ohtake

**Affiliations:** 1 Faculty of International Studies, Meiji Gakuin University, Yokohama/Tokyo, Japan; 2 Center for Infectious Disease Education and Research (CiDER), Osaka University, Osaka, Japan; 3 School of Economics and Management, University of Hyogo, Hyogo, Japan; 4 Center for Infectious Disease Education and Research (CiDER), Graduate School of Economics, Osaka University, Osaka, Japan

**Keywords:** COVID-19, Handwashing education, Hygiene, Reciprocity, Shrines

## Abstract

**Background:**

The role of social ties, other-regarding preferences, and cultural traits in boosting community resilience and minimizing citizens’ vulnerability to crises such as COVID-19 is increasingly being recognized. However, little is presently known about the possible routes through which such personal preferences and cultural norms pertinent to social behaviors are *formulated*. Thus, in this paper, factors that can be potentially associated with individuals to self-regulate strict hand hygiene practices before the pandemic, during the state of emergency, and after the state of emergency was lifted in Japan are investigated. Focus is given to the handwashing education in primary school, a cultural practice originating from the old Shinto tradition, and individuals’ reciprocal inclinations. As people in Japan are known to be highly conscious of hygiene in all aspects of their daily life and are less likely to contract an infection, evidence obtained in this specific context could contribute to the better understanding of individuals’ health-related behaviors in general, and during crises in particular.

**Methods:**

Using the data derived from a four-wave nationwide longitudinal online survey, we examined the extent to which elementary school education, childhood cultural experiences at shrines, and individual other-regarding preferences are associated with self-regulating hand hygiene practices prior to the pandemic and people’s efforts to comply with the government-imposed measures aimed at preventing the spread of COVID-19 infection during the state of emergency. We also investigated the long-term trends in the relationships among these factors (i.e., after the abolishment of the state of emergency) using panel data.

**Results:**

Our findings reveal that childhood education and cultural experiences related to handwashing practices, as well as reciprocal inclinations, are significantly associated with Japanese attitudes toward personal hygiene (beyond handwashing practices) prior to, during, and after the state of emergency. In recognition of the possible effects of recall bias and measurement errors, several important attempts to mitigate these issues were made to strengthen the value of our findings.

**Conclusions:**

The importance of school education received during childhood, as well as culture and other-regarding preferences, in the individual attitudes toward hand hygiene in adulthood highlighted in this study contributes to the better understanding of the role that these factors play in the variations in *voluntary* compliance with strict hand hygiene practices before and during an uncertain and prolonged crisis.

**Supplementary Information:**

The online version contains supplementary material available at 10.1186/s12889-022-14012-z.

## Background

Since the COVID-19 outbreak, numerous studies have been conducted on the capacity of healthcare systems [1, 2], population density [3, 4], and government restrictions on social gatherings [5, 6] as potential explanations for considerable variations in the consequences of the pandemic for communities, states, and nations. Several authors have also emphasized the importance of social ties, other-regarding preferences, and cultural traits in overcoming the pandemic, which were in some cases found to be more influential than the public policies implemented by governments and public health institutions, especially when these are insufficiently developed [7, 8].

For example, Alfaro et al. [9] established that government-imposed measures are less relevant for individuals that are more patient and altruistic, or exhibit less negative reciprocity, confirming the importance of social and other-regarding preferences in individuals’ response to the pandemic, their cooperative attitudes, and mobility decisions. Furthermore, several authors examined cultural variations and norms as potential determinants of social mobility change, focusing on distances between interacting people and social contact frequencies [10], and Schwartz’s cultural value orientations that are significantly associated with “hierarchy” [11].

While social and cultural traits are increasingly recognized as powerful interventions that boost community resilience and minimize citizens’ vulnerability to crises, little is presently known about factors that could potentially *underlie* such personal preferences and cultural norms pertinent to social behaviors. In this work, we focus on handwashing education and cultural experiences in childhood as we argue that individuals’ attitudes toward personal hygiene are formed in this crucial period [12], and may thus reflect their self-regulated hand hygiene behaviors during the COVID-19 pandemic. As we conducted the estimations based on our participants’ retrospective responses regarding their childhood education and experiences, our results may be impacted by recall bias as well as measurement errors. While it would not be possible to completely solve these problems with the data and empirical methods used for this study, as we made several important attempts to mitigate the possible biases and errors, we believe that the association between school education and experiences in childhood and hand hygiene practices in adulthood established in this work could be useful for better understanding the health behavior of general public.

For example, our empirical results indicate that handwashing education in elementary school could potentially relate to hygiene behaviors in adulthood, and that, even though some cultural factors are invisible, they can implicitly inform the way we conduct our daily life. Furthermore, in line with pertinent literature [13, 14], we demonstrated the positive role of reciprocity, which is interrelated with the cultural traits and school education, in various hand hygiene practices, suggesting its significant implications for behaviors during crises. While the aforementioned limitations implicit in our study design preclude formation of any causal inferences, our findings could still serve as a valuable foundation for future efforts to identify determinants that drive citizens to voluntarily avoid social contact, strictly comply with hand hygiene practices, and adopt appropriate self-care measures. Findings yielded by such studies might obviate the need for drastic public health protocols, thereby reducing the economic and social burden of any future pandemics.

### Hand hygiene education in elementary schools and ritual customs at shrines/temples

As Japanese are well known for their longest life expectancy at birth, considerable attempts have been made to identify the factors that contribute to their longevity and good health. Ikeda et al. [15] are of view that, as Japanese people are highly conscious of hygiene (especially regular handwashing) in all aspects of their daily life, they are less likely to contract an infection. They further argue that these attitudes stem from a complex interaction of culture, education, climate, and environment. Hence, we first review the Japanese educational policies regarding hand hygiene practices, the cultural traits, and other-regarding preferences originating from the old Shinto tradition, as we hypothesize that these factors, while being interrelated, contribute to Japanese citizens’ voluntary self-restrictions and high compliance with hand hygiene regulations.

In Japan, emphasis on personal hygiene starts in nursery school (when children are aged 0 − 3 years) and kindergarten (3 − 6 years), and continues through elementary (6 − 12 years) and secondary school (12 − 18 years) (for the Japan’s educational system, refer to [16]). To promote healthy mental and physical development of schoolchildren, the School Lunch Act *(Gakko Kyusyoku Ho* in Japanese) was enacted in 1954 and was expanded to all compulsory education in 1956 [17]. After several rounds of revision to Elementary School Teaching Guide for the Japanese Course of Study (*Syougakko Gakusyu Sidou Yoryou Kaisetsu* in Japanese), the school lunch program became an integral part of the school curriculum in 1968 to ensure that all children are educated on the importance of hygiene [18]. As a part of this ongoing initiative, children are trained to serve food and clean up after meals while respecting all hygiene practices, including wearing caps and white aprons, and to comply with table manners (which includes washing their hands before each meal).

**Fig. 1:**
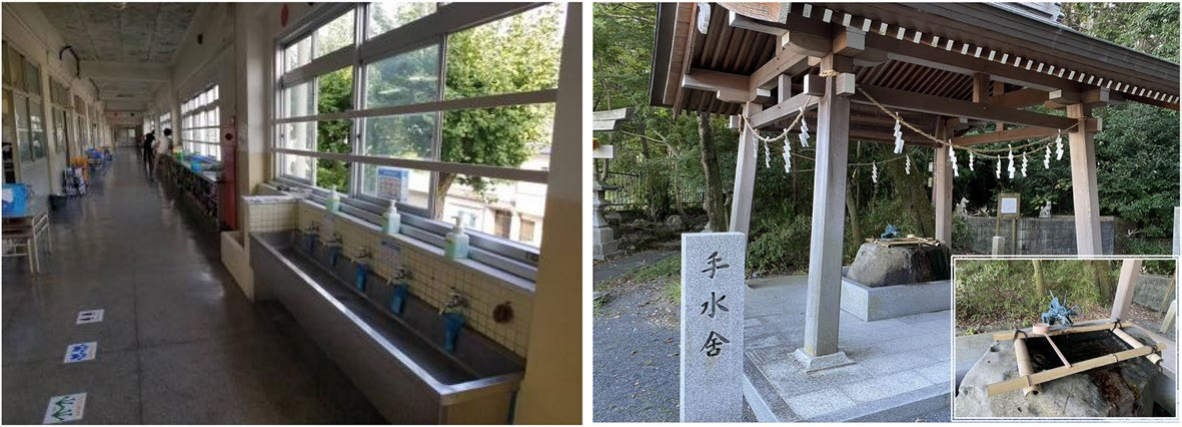
Photos of Handwashing Stands at an Elementary School and at a Shrine. Note: The photo on the left shows an elementary school equipped with handwashing facilities in the corridors on each floor of the building, which usually comprise of a row of cold-water taps each with a bar of soap in a mesh bag or a liquid hand soap. The photo on the right shows handwashing facilities that are common in shrines/temples in Japan (for more details, see the additional photo in the right bottom corner) where visitors are expected to clean their hands and mouth to purify their body and mind before any prayers. Both photos are taken by the authors

In addition, all pupils are instructed that handwashing is the single most important factor in preventing community-acquired infection and is thus part of normal duty of care. For this purpose, regular handwashing practices are instructed by teachers who monitor students’ adherence to these measures. To aid with this process, elementary schools are equipped with handwashing facilities, such as long sinks in the corridors on each floor of the school building (as shown in Fig. 1), as well as near the toilets and in the schoolyard. Students wash their hands with soap before and after lunch, as well as after science experiments and bathroom breaks.

**Fig. 2:**
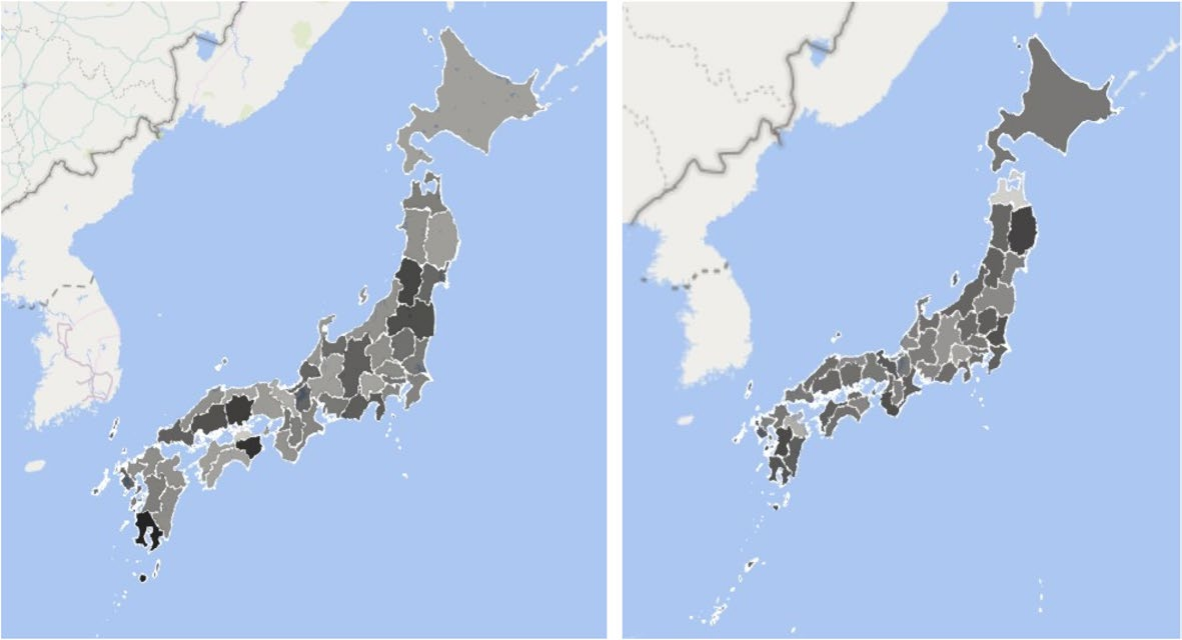
Handwashing Education in Elementary Schools and Reciprocity by Prefecture. Note: In the figure on the left, percentages of respondents in different prefectures (ranging from Hokkaido to Okinawa) who received handwashing education at their elementary school are depicted on the map of Japan (darker colors indicate schools with regular teacher-supervised handwashing in the given prefecture; range = 0.1─0.38). In the figure on the right, the degree of reciprocity is depicted on the map of Japan (darker colors indicate a higher mean value of reciprocity; range = 0.18─0.70)

However, as evident from Fig. 2, the stringency of teachers’ involvement (which could be related to school discipline policy and educational philosophy) differs across schools and prefectures, where a darker color indicates that a greater percentage of respondents in the given prefecture received strict handwashing education at their elementary school. Given that students are given health handouts to take home from school that state the importance of washing their hands frequently, along with going to bed early, exercising regularly, and gargling as soon as they get home, educational policy is expected to have wide-ranging implications for their attitudes toward hygiene throughout their lives. The handwashing education in Japanese elementary schools is further strengthened by the engagement of the Japan Soap and Detergent Association (JSDA) in awareness-raising activities to deepen consumers’ appreciation of the importance of handwashing [19]. This initiative commenced in 1950 with distribution of free soap to elementary schools. The JSDA has also released a handmade soap recipe and has recommended that this activity be instructed by teachers at schools.

In addition to the role of education, we examine the role played by culture in regular hand hygiene practices by considering the handwashing experiences in shrines/temples participants visited during childhood. Shinto shrines and Buddhist temples are broadly and intimately engaged in the life of Japanese. Even though Japanese do not consider themselves particularly religious, both shrines and temples are woven into the fabric of everyday life through Japanese traditional weddings at shrines and funerals at temples. Before praying at Japanese shrines/temples, it is customary for visitors to clean their hands (and mouth) to purify their body and mind (as shown in Fig. 1). The possible implications of invisible cultural factors for personal hygiene routine are found in relation to the religious norms and beliefs in other countries [20, 21], where purification rituals and practices are believed to translate into more stringent hygiene practices in other parts of their daily life. Similarly, given the importance of tradition in the daily life of Japanese, we posit that frequent exposure and adherence to the ritual customs for purification early in one’s life could be associated with a greater perceived value of hygiene, which should implicitly increase adherence to regular handwashing practices on a daily basis.

### Reciprocity interrelated with school education and cultural experiences

Fehr and Schmidt [22] argued that social and economic activities cannot be fully explained by the self-interest model, and that other-regarding preferences should be considered as a motivation for individuals’ economic decisions and social interactions (see also [23, 24]). Due to the recent COVID-19 pandemic, all aspects of social life had to be adjusted in order to protect one’s health and that of others. In this context, according to Alfaro et al. [9], it is expected that the pro-social attitudes—such as altruism, patience, and cooperation—as well as attitudes toward reciprocity, would play a role in individual decisions and behaviors during the recent COVID-19 pandemic, and thus their voluntary adherence to public health policies. Viens et al. [14] also emphasized that reciprocity is crucial in this context, as it demands an appropriate balancing of the benefits and burdens of social cooperation. Therefore, as reciprocal inclinations, or other regarding preferences, could be associated with variations in people’s adherence to the public hygiene expectations and regulations, we focus on the direct links of reciprocity with hand hygiene practices during the pandemic. Furthermore, we also examine the role of reciprocity in association with distinct cultural traits revolving shrines/temples in a country and the country’s elementary school curriculum on students’ hand hygiene practices.

This decision is guided by the findings reported by Ito et al. [25] suggesting that, as shrines are engaged in community festivals, people living close to shrines are more likely to be exposed to the local community practices. Thus, this is likely to relate to the other-regarding preferences (reciprocal tendencies in particular) at the individual level. Hence, we posit that adults that lived near shrines as children are more likely to be reciprocal because their concern for others would be cultivated by the community-focused experiences surrounding shrines. Furthermore, hand hygiene education received in school could enlighten students on the importance of caring for others by preventing the spread of infections [26], which would likely become an integral aspect of their social interactions during their lifetime. Hence, we examine the interaction of reciprocal inclinations with the school education and cultural experiences in childhood in addition to investigating the direct links of the aforementioned factors with hand hygiene practices separately.

## Methods

### Survey design

The data for this study was obtained via online surveys conducted in 2020 by MyVoice.Com under the authors’ guidance. We extracted participants throughout Japan using their monitors, such that the individuals’ gender and age (20–69 years) ratios would be equal to the national population. We verified that our sample is comparable with respect to the distribution of key individual characteristics to the Japanese population based on the findings reported by the government and the United Nations: household income and gender ratio by age group [27, 28], years of education [29], and distribution of population by prefecture [30]. In the initial survey, conducted from April 28th to 30th, the participants (*n* = 6,050) were asked about their hand hygiene behavior before and during the pandemic. Three further waves were conducted from May 8th to 13th (*n* = 5,664), from June 8th to 12th (*n* = 4,846), and July 28th to August 3rd (*n* = 4,501) (for further information, see [31]).^1^

As Japanese government declared the state of emergency on April 7th in seven major prefectures, which was expanded to have a nationwide effect on April 16th, and was subsequently gradually lifted until its complete abolishment on May 25th, all four survey waves were conducted when the state of emergency was either imposed (1^st^ and 2^nd^ waves) or lifted (3^rd^ and 4^th^ waves) *across* the nation.^2^ As a result, we used the data gathered during the first wave to investigate participants’ hand hygiene behavior before COVID-19 (using retrospective questions) and during the first state of emergency in Japan, while we relied on the fourth wave to investigate the long-term trends in the general hygiene practices during crises. The fourth survey wave (end of July/early August) coincided with a nationwide surge in coronavirus infections following the abolishment of the state of emergency, which might have been associated with a significant difference in respondents’ attitudes compared to the earlier waves.

### Outcome variables: handwashing and hygiene practices

As regular handwashing and mask wearing in public, as well as minimizing all unnecessary social contact and travel, are seen as the most effective means of curbing the COVID-19 spread [32], we use two sets of outcome variables to investigate the factors that are associated with these preventive measures. The first set relates to the handwashing practices in four situations when this is deemed most essential, namely after coming home, after meals, and after toilet use (separately for urination and defecation).^3^ Survey respondents were asked to report on these handwashing practices in all survey waves and were explicitly asked to indicate if they use soap (coded as 1, and 0 otherwise) in each of these instances, as we assumed that childhood experiences related to handwashing at shrines (where soap is not used) and the handwashing education at elementary school (where usage of soap is emphasized) could differently relate to respondents’ handwashing practices later in life. We also considered the average score related to the aforementioned handwashing practices.

Our second set of outcome variables relates to the hygiene practices introduced by the Japanese government to prevent the spread of respiratory infections and lower the risk of COVID-19 transmission, as advised by the WTO [33]. As adherence to social distancing rules is expected to vary among individuals, we measured individual efforts to avoid physical contact: (i) by wearing a mask and avoiding handshakes; (ii) by preventing spread of infections through touch; (iii) by minimizing social contact in public; and (iv) by improving adaptive coping responses such as staying at home and self-care to maintain health and prevent disease [34, 35]. As most of these practices were strictly enforced during the pandemic, we only asked respondents to state their compliance with the aforementioned hygiene behaviors during the week immediately prior to taking part in each survey wave (i.e., this dataset does not include hygiene practices before COVID-19). When completing this section of the survey, participants were required to rate fifteen hygiene practices on a 0 − 7 scale (for details, see Table 1), where the higher values indicate the respondent’s stronger agreement with the given statement.

**Table 1:** Descriptive Statistics of Outcome Variables

**Outcome Variables**	In April (*n* = 6050)		In July/August (*n* = 4501)	
	Mean	Standard Deviation (S.D.)	Mean	S.D
**Handwashing Practices with Water (0/1)**				
I wash my hands after coming home from outside	0.953	0.213	0.957	0.203
I wash my hands before each meal	0.854	0.353	0.873	0.333
I wash my hands at the workplace or in other public spaces after toilet use (urination)	0.957	0.202	0.955	0.207
I wash my hands at the workplace or in other public spaces after toilet use (defecation)	0.958	0.200	0.951	0.216
Average of the aforementioned four practices	0.931	0.191	0.934	0.177
**Handwashing Practices with Soap (0/1)**				
I wash my hands after coming home from outside	0.833	0.373	0.810	0.393
I wash my hands before each meal	0.585	0.493	0.566	0.496
I wash my hands at the workplace or in other public spaces after toilet use (urination)	0.633	0.482	0.597	0.491
I wash my hands at the workplace or in other public spaces after toilet use (defecation)	0.706	0.456	0.687	0.464
Average of the aforementioned four practices	0.689	0.373	0.665	0.378
**Hygiene Practices (0–7)**				
*Wearing a mask*				
When coughing or sneezing, I place a mask or handkerchief over my mouth ("cough etiquette")	5.249	2.460	5.380	2.378
I always wear a mask when talking to someone	5.061	2.322	5.443	2.052
I always wear a mask when going out	5.798	2.092	6.032	1.827
*Preventing the spread of infection through hand contact*				
I practice gargling and frequently wash my hands, and disinfect my hands and fingers with alcohol	5.968	1.783	5.978	1.700
I try to avoid shaking hands	5.530	2.406	5.732	2.181
I use cashless payment methods (credit cards, electronic fund transfers, etc.) instead of cash	4.500	2.559	4.654	2.419
*Preventing occurrence of "clusters"*				
I talk with others via phone or video call whenever possible	4.121	2.773	3.960	2.730
I designate one person to do the shopping or go out in a small group during times when stores are not crowded	5.046	2.207	4.954	2.163
I use takeout or home delivery services instead of going to restaurants	3.865	2.937	3.559	2.760
I use delivery or mail-order services for larger purchases	3.721	2.848	3.682	2.749
*Self-care during COVID-19 pandemic*				
I avoid going out when I feel unwell	4.986	2.656	5.146	2.524
I try to stay home as much as possible even if I am not ill	5.702	1.989	5.189	2.197
I try to avoid touching my face	4.088	2.394	4.289	2.331
I try to get plenty of rest and sleep	5.526	1.901	5.304	1.922
I try to eat nutritious foods	4.755	2.052	4.673	2.058
Average of the aforementioned fifteen practices	4.928	1.511	4.932	1.467

### Empirical framework (1) regarding education and cultural factors

We first examined the extent to which handwashing education in elementary school and living environment (residing near shrines/temples) during childhood are associated with the hand hygiene attitudes and behaviors later in life (${\mathrm{H}}_{\mathrm{i}}$) by employing the Ordinary Least Squares (OLS) based on the following model:

1 $${\mathrm{H}}_{\mathrm{i}}={\alpha }_{0}+{\alpha }_{1}{\mathrm{Education}}_{\mathrm{i}}+{\alpha }_{2}{\mathrm{Residence}}_{\mathrm{i}}+{\mathrm{X}}_{\mathrm{i}}\upgamma +{\upvarepsilon }_{\mathrm{i}}$$

where i indexes individuals, ${\mathrm{X}}_{\mathrm{i}}$ is the vector of controls that are expected to relate to ${\mathrm{H}}_{\mathrm{i}}$, and ${\upvarepsilon }_{\mathrm{i}}$ is an unobserved component relating to ${\mathrm{H}}_{\mathrm{i}}$. We assumed that E [${\upvarepsilon }_{\mathrm{i}}$] = 0, while our goal was to examine the value of parameters ${\alpha }_{1}$ and ${\alpha }_{2}$. ${\mathrm{H}}_{\mathrm{i}}$ relates to the two sets of outcome variables regarding handwashing and hygiene practices. Specifically, we examined the role of ${\mathrm{Education}}_{\mathrm{i}}$ and ${\mathrm{Residence}}_{\mathrm{i}},$ which respectively pertain to the handwashing education received in elementary school and residing near shrines in childhood (hereafter, education/residence). All aforementioned variables were constructed as dummies based on the participants’ responses to the following statements: (1) “Everyone in my class at elementary school was supervised by teachers to ensure that they washed their hands in turn before lunch and after physical education”; (2) “There was a handmade soap making class in my elementary school”; (3) “There were shrines near my house or along the school route in my childhood”; and (4) “There were temples near my house or along the school route in my childhood” (hereafter, residence “near” shrines/temples indicates that shrines/temples were in close proximity to their home/route to school and were constantly in line of sight).

Other confounding variables consist of gender, birth cohort (age), prefecture where a respondent lived in the first year of elementary school, and prefecture of the current residence (all of which were dummy variables), level of educational attainment (ranging from 1 equivalent to elementary school level, to 11 assigned for a doctoral degree), being married (= 1), number of children, sharing a household with a person aged ≥ 65 (= 1), and having a child attending elementary school (assuming that, comparatively, handwashing and hygiene practices are more rigorously maintained in elementary school; = 1), along with other dummies for different educational levels of children (e.g., preschool and secondary). Note that the generational and regional differences in handwashing and hygiene education at the elementary school level are implicitly controlled by birth cohort and prefecture fixed effects. We also controlled for some behavioral factors related to the hand hygiene practices (e.g., [9]), namely altruistic behavior (intention to donate [1 − 5] and volunteer participation [1 − 6]); risk-taking inclinations (0 − 10); and time discounting preference/delayed gratification (0 − 10). For labor variables, we used information on household income, occupation, employment status, and whether the respondent is able/willing to work remotely, all of which were constructed as dummies (for more details, see Appendix 3).^4^

### Empirical framework (2) regarding ritual customs and reciprocity

As our analyses also probed into the role of childhood exposure to handwashing rituals at shrines and current reciprocal inclinations (hereafter, customs/reciprocity) in individual behavior during COVID-19, we asked the participants to select one of the following responses to the question “When you were a child, how often did you wash your hands in shrines and/or temples?”: “I did not wash at all” (base); “I washed my hands only a few times”; “I washed my hands most of the time”; “I always washed hands”), while, albeit undocumented, the choices corresponding to “I have never visited a shrine/temple” and “Do not remember” were also controlled for. The “reciprocity” variable was constructed based on the ratings on a 5-point scale (with 1 denoting “strongly disagree” and 5 “strongly agree”) of the following statement: “If others do me a favor, I am prepared to return it.”

Following Tu et al. [36], we distinguished between two different, but complementary, association with the outcome variables: (i) education/residence associated with hand hygiene practices directly, as shown in Eq. (1); and (ii) education/residence associated with hand hygiene practices, via their association with customs/reciprocity, which are in turn correlated to high compliance with hand hygiene practices (i.e., education/residence → customs/reciprocity → hand hygiene practices). According to Tu et al. [36], the model provided by Eq. (1) would not be suitable for testing these pathways with adjustments for these concomitant variables, because concomitant variables lie on the direct path between the outcome (hand hygiene practices) and the exposure (education/residence). Rosenbaum [37] also emphasized that the inappropriate use of statistical adjustments for confounders is an important source of potential bias in observational studies. Hence, we first estimated Eq. (1) without concomitant variables—customs/reciprocity.

We considered customs/reciprocity as concomitant variables because the handwashing education received at school is expected to relate to the children’s handwashing habits in other aspects of their lives, including purification rituals at local shrines. Furthermore, adults that lived near shrines as children would also be expected to visit shrines more frequently than those residing further away, and would be more exposed to the ritual customs at shrines. The possible links between education/residence and reciprocity were explained in the Background section (under the “Reciprocity Interrelated with School Education and Cultural Experiences” heading), and the links between education/residence and customs/reciprocity are also confirmed by positive correlations yielded by our analyses, examined in detail in the Discussion section.

To investigate the link between outcome variables and these concomitant variables (customs/reciprocity), which are plausible surrogates for clearly relevant confounding variables [37], even after adjustments for confounding variables (education/residence), we developed the following model:

2 $${\mathrm{H}}_{\mathrm{i}}={\upbeta }_{0}+{\upbeta }_{1}{\mathrm{Education}}_{\mathrm{i}}+{\upbeta }_{2}{\mathrm{Residence}}_{\mathrm{i}}+{\upbeta }_{3}{\mathrm{Ritual}\_\mathrm{Customs}}_{\mathrm{i}}+{\upbeta }_{4}{\mathrm{Reciprocity}}_{\mathrm{i}}+{\mathrm{X}}_{\mathrm{i}}\uptheta +{\upvarepsilon }_{\mathrm{i}}$$

### Compliance to hygiene public policy

The first survey wave probed into the respondents’ compliance with handwashing practices before COVID-19 and in April separately, while in the fourth wave these same questions related to the degree of compliance in July/August when the state of emergency had been lifted. We examined the changes in the associations of education, cultural factors, and reciprocity with current hand hygiene practices from April (first wave) to July/August (fourth wave). Then, the responses provided in the first wave were adjusted for those in the fourth wave to establish the extent to which these factors contributed to prompting the compliance with the handwashing and hygiene practices from April to July/August.

## Results

### Descriptive statistics

The descriptive statistics related to the outcome variables (${\mathrm{H}}_{\mathrm{i}}$) measured as a part of the first and fourth survey waves (in April and July/August) are presented in Table 1. When reporting on their handwashing practices in April, 83%, 59%, 63%, and 71% of the sample stated that they washed their hands with soap after coming home, before meals, after urination, and after defecation, respectively, while these percentages increased to approximately 85 − 95% for washing with water only. As shown in Appendix 1 and Appendix 2, the mean values in the subsequent waves are higher than those pertaining to the first wave and pre-COVID period (only for handwashing practices), which was expected, suggesting that high risk of infection prompted the respondents to increase their handwashing frequency and hygiene practices. Exceptions are observed in adherence to social contact (e.g., talking on the phone, using a takeout service) and self-care management (e.g., staying at home even if not ill, getting plenty of rest), which decreased. These are reasonable results considering that the fourth wave was conducted when the state of emergency that enforced social distancing was lifted.

As indicated in Appendix 3, 23% and 12% of the respondents indicated that, in school, students were supervised by teachers to ensure that they washed their hands and there was a handmade soap class, respectively, while 49% and 37% of the sample lived near shrines and temples in childhood, respectively. Among 59.6% of the respondents who had visited shrines/temples in the past, hand washing was a common practice (i.e., they indicated that they washed hands “most of the time” or “always”). Finally, the mean value of 3.96 for reciprocity was obtained for the full sample, suggesting that the respondents on average concurred with the statement.

It is also worth noting that 26.7% of the sample shared household with a person aged ≥ 65, who we focus on in this study given that the elderly are most vulnerable to the most severe forms of COVID-19 infection. We also considered having children attending elementary school relevant (5.55%), as handwashing education at elementary school (as the key phenomenon of interest of this study) may be associated with families’ willingness to adhere to these practices more strictly to align with the instructions children receive as a part of the school curriculum.^5^ We also constructed dummies for all labor variables listed in Appendix 3, and were particularly interested in respondents’ ability/willingness to work remotely at each survey wave. Thus, it is noteworthy that, at the time of the first wave (conducted in April), 61.4% of individuals that were employed indicated that remote work was not available.

### Handwashing practices before the COVID-19 pandemic

As can be seen from Table 1, approximately 90% (70%) of Japanese citizens regularly washed their hand with water (using soap) in April. In Table 2, we focus on the factors that are associated with these behaviors. The results reported in Columns 1 − 5 and 6 − 10 relate to handwashing with water, and using soap, respectively. Moreover, Panel A pertains to Eq. (1) while Panel B relates to Eq. (2).

**Table 2:** Handwashing Practices Before COVID-19

	Handwashing Practices (Before COVID-19) with Water					Handwashing Practices (Before COVID-19) with Soap				
	Washing hands after coming home	Washing hands before eating	Washing hands after toilet use (urination)	Washing hands after toilet use (defecation)	Average of four items	Washing hands after coming home	Washing hands before eating	Washing hands after toilet use (urination)	Washing hands after toilet use (defecation)	Average of four items
	(1)	(2)	(3)	(4)	(5)	(6)	(7)	(8)	(9)	(10)
**Panel A: Eq. (****1****) Adjustments for childhood education/residence as below, as well as demographic/ behavioral/labor/age and prefecture dummies**										
*Elementary School Education*										
Everyone in my class was supervised by teachers to ensure that they washed their hands in turn	0.0380***	0.0380***	0.0143*	0.0160**	0.0346***	0.0502***	0.1006***	0.0907***	0.0897***	0.0828***
	(0.011)	(0.011)	(0.008)	(0.008)	(0.008)	(0.017)	(0.018)	(0.018)	(0.017)	(0.014)
There was a handmade soap making class	0.0403***	0.0403***	0.0216**	0.0113	0.0421***	0.0847***	0.1170***	0.1108***	0.0696***	0.0955***
	(0.014)	(0.014)	(0.009)	(0.009)	(0.009)	(0.020)	(0.022)	(0.022)	(0.021)	(0.017)
*Childhood Residential Area Near Shrines/Temples*										
There were shrines near my house or along the school route	0.0091	0.0091	0.0156**	0.0123*	0.0062	-0.0096	-0.0327*	-0.0236	-0.0013	-0.0168
	(0.011)	(0.011)	(0.007)	(0.007)	(0.007)	(0.016)	(0.017)	(0.017)	(0.017)	(0.014)
There were temples near my house or along the school route	-0.0078	-0.0078	-0.0026	-0.0021	-0.0028	0.0005	0.0172	0.0089	0.0110	0.0094
	(0.011)	(0.011)	(0.007)	(0.007)	(0.007)	(0.016)	(0.017)	(0.017)	(0.017)	(0.014)
**Panel B: Eq. (****2****) Adjustments for handwashing experiences at shrines/temple and reciprocity as below in addition to those in Panel A**										
*Elementary School Education*										
Everyone in my class was supervised by teachers to ensure that they washed their hands in turn	0.0305***	0.0305***	0.0075	0.0087	0.0267***	0.0418**	0.0890***	0.0776***	0.0788***	0.0718***
	(0.011)	(0.011)	(0.008)	(0.007)	(0.008)	(0.017)	(0.018)	(0.018)	(0.017)	(0.014)
There was a handmade soap making class	0.0359***	0.0359***	0.0192**	0.0087	0.0378***	0.0783***	0.1071***	0.1007***	0.0635***	0.0874***
	(0.014)	(0.014)	(0.009)	(0.009)	(0.009)	(0.020)	(0.021)	(0.021)	(0.021)	(0.017)
*Childhood Residential Area Near Shrines/Temples*										
There were shrines near my house or along the school route	0.0112	0.0112	0.0115	0.0080	0.0057	-0.0029	-0.0244	-0.0154	0.0056	-0.0093
	(0.011)	(0.011)	(0.007)	(0.007)	(0.007)	(0.016)	(0.017)	(0.017)	(0.017)	(0.014)
There were temples near my house or along the school route	-0.0089	-0.0089	-0.0039	-0.0035	-0.0044	0.0003	0.0151	0.0074	0.0104	0.0083
	(0.011)	(0.011)	(0.007)	(0.007)	(0.007)	(0.016)	(0.017)	(0.017)	(0.017)	(0.014)
*Childhood Handwashing Experiences at Shrines/Temples*										
Never washed hands (Base)										
Washed hands only a few times	0.0290**	0.0290**	0.0231**	0.0227**	0.0255**	0.0009	0.0170	0.0123	0.0469**	0.0193
	(0.015)	(0.015)	(0.010)	(0.010)	(0.010)	(0.022)	(0.023)	(0.023)	(0.023)	(0.018)
Washed hands most of the time	0.0644***	0.0644***	0.0281***	0.0284***	0.0490***	0.0651***	0.0976***	0.1016***	0.1033***	0.0919***
	(0.014)	(0.014)	(0.009)	(0.009)	(0.009)	(0.021)	(0.022)	(0.022)	(0.021)	(0.017)
Always washed hands	0.0778***	0.0778***	0.0304***	0.0312***	0.0672***	0.0986***	0.1617***	0.1607***	0.1228***	0.1360***
	(0.015)	(0.015)	(0.010)	(0.010)	(0.010)	(0.022)	(0.024)	(0.024)	(0.023)	(0.019)
*Reciprocity*										
Reciprocity	0.0082	0.0082	0.0292***	0.0299***	0.0140***	0.0090	-0.0174**	0.0016	0.0218***	0.0038
	(0.005)	(0.005)	(0.003)	(0.003)	(0.004)	(0.008)	(0.008)	(0.008)	(0.008)	(0.007)
Observations	6,050	6,050	6,050	6,050	6,050	6,050	6,050	6,050	6,050	6,050

Results yielded by Eq. (1) are presented in Panel A. Note that in Panel A, childhood handwashing experiences at shrines/temples and reciprocity (customs/reciprocity) are intentionally excluded from these findings because these two sets of variables are considered as concomitant variables which are a plausible surrogate for more immediately relevant confounding variables (education / residence). Individual characteristics, behavioral factors, labor-related variables, and age/prefecture dummies are also controlled for (for more detailed information, see table A3). Standard errors are given in parentheses. ***, **, and * indicate *p* < .01, *p* < .05, and *p* < .1

As shown in Panel A, the elementary school education is significantly associated with the regular handwashing practices with water and soap in both cases,^6^ while living near shrines (where soap is not used) in childhood is positively associated with current handwashing practices with *water* (similar results are also reported in Appendix 4). The results reported in Panel B further reveal that individuals who washed their hands at shrines/temples (even if they did so rarely) tend to wash their hands with water and soap more regularly than those who did not wash their hands in shrines/temples at all in childhood. Moreover, high reciprocal inclinations tend to be positively associated with regular handwashing practices. In sum, both confounding (education/residence in Panel A) and concomitant (customs/residence in Panel B) variables are significant factors of handwashing practices.

It should be noted that, in Panel B, we mainly focus on customs/reciprocity that were obtained after controlling for education/residence during childhood (along with other controls), while education/residence are reported in Panel B for comparison purposes. The results indicate that these confounding variables become less or not significant and the magnitudes of the coefficients become smaller with the adjustment of the concomitant variables in Panel B. This suggests that as hypothesized, the concomitant variables lie on the direct path between confounding and outcome variables.

Moreover, females, more educated and married individuals, parents with fewer children regardless of their age, those who share a household with a person aged ≥ 65, and those that have children attending elementary school are more likely to wash their hands with both water and soap (undocumented).^7^ Similarly, those willing to make charitable donations, which can be interpreted as a sign of altruism, were more likely to adopt appropriate handwashing practices, as were more risk averse individuals.

### Hand hygiene practices during and after the COVID-19-related state of emergency

In Table 3, we report the results related to the first survey wave, when the state of emergency was first declared, and the fourth survey wave, when the state of emergency was first eliminated, respectively. Due to the space constraints, only the results of the *average* of four items for handwashing with water and soap, and those of the *average* of fifteen items regarding self-regulating hygiene practices (such as wearing a mask, avoiding social gatherings, and self-care) are reported (specific cases are provided in Appendices 4 and 5).

**Table 3:** Handwashing and Hygiene Practices During COVID-19

	Handwashing with water (Average of four items)			Handwashing with soap (Average of four items)			Hygiene practices (Average of fifteen items)		
Time periods	April	July/August (J/A)	J/A(controlling for April)	April	July/August (J/A)	J/A(controlling for April)	April	July/August (J/A)	J/A(controlling for April)
	(1)	(2)	(3)	(4)	(5)	(6)	(7)	(8)	(9)
**Panel A: Eq. (****1****) Adjustments for childhood education/residence as below, as well as demographic/ behavioral/labor/age and prefecture dummies**									
*Elementary School Education*									
Everyone in my class was supervised by teachers to ensure that they washed their hands in turn	0.0256***	0.0191***	0.0124*	0.0666***	0.0608***	0.0164	0.3159***	0.2871***	0.1084**
	(0.007)	(0.007)	(0.007)	(0.015)	(0.015)	(0.012)	(0.058)	(0.059)	(0.049)
There was a handmade soap making class	0.0278***	0.0203**	0.0122	0.0817***	0.0833***	0.0294**	0.1864***	0.1452**	0.0388
	(0.009)	(0.009)	(0.009)	(0.019)	(0.019)	(0.014)	(0.071)	(0.072)	(0.060)
*Childhood Residential Area Near Shrines/Temples*									
There were shrines near my house or along the school route	0.0030	0.0008	0.0015	-0.0074	-0.0096	-0.0047	0.0071	0.0510	0.0477
	(0.007)	(0.007)	(0.007)	(0.014)	(0.015)	(0.011)	(0.055)	(0.056)	(0.046)
There were temples near my house or along the school route	0.0035	-0.0007	-0.0011	0.0033	0.0070	0.0045	0.0982*	0.0263	-0.0284
	(0.007)	(0.007)	(0.007)	(0.014)	(0.015)	(0.011)	(0.055)	(0.056)	(0.046)
**Panel B: Eq. (****2****) Adjustments for handwashing experiences at shrines/temple and reciprocity as below in addition to those in Panel A**									
*Elementary School Education*									
Everyone in my class was supervised by teachers to ensure that they washed their hands in turn	0.0200***	0.0138*	0.0084	0.0555***	0.0490***	0.0123	0.2617***	0.2435***	0.0968**
	(0.007)	(0.007)	(0.007)	(0.015)	(0.015)	(0.012)	(0.058)	(0.059)	(0.049)
There was a handmade soap making class	0.0238***	0.0166*	0.0095	0.0739***	0.0745***	0.0261*	0.1500**	0.1144	0.0294
	(0.009)	(0.009)	(0.009)	(0.019)	(0.019)	(0.014)	(0.071)	(0.072)	(0.060)
*Childhood Residential Area Near Shrines/Temples*									
There were shrines near my house or along the school route	0.0027	0.0014	0.0016	-0.0023	-0.0040	-0.0025	0.0138	0.0639	0.0567
	(0.007)	(0.007)	(0.007)	(0.014)	(0.015)	(0.011)	(0.055)	(0.056)	(0.047)
There were temples near my house or along the school route	0.0017	-0.0019	-0.0021	0.0013	0.0045	0.0033	0.0824	0.0174	-0.0281
	(0.007)	(0.007)	(0.007)	(0.014)	(0.015)	(0.011)	(0.055)	(0.055)	(0.046)
*Childhood Handwashing Experiences at Shrines/Temples*									
Never washed hands (Base)									
Washed hands only a few times	0.0242***	0.0080	0.0058	0.0220	0.0032	-0.0118	0.2240***	0.0704	-0.0533
	(0.009)	(0.009)	(0.009)	(0.019)	(0.020)	(0.015)	(0.074)	(0.075)	(0.063)
Washed hands most of the time	0.0447***	0.0352***	0.0256***	0.0996***	0.0809***	0.0153	0.2981***	0.2319***	0.0664
	(0.009)	(0.009)	(0.009)	(0.018)	(0.019)	(0.014)	(0.071)	(0.072)	(0.060)
Always washed hands	0.0470***	0.0415***	0.0308***	0.1100***	0.1201***	0.0474***	0.4882***	0.3642***	0.0919
	(0.010)	(0.010)	(0.010)	(0.020)	(0.020)	(0.016)	(0.076)	(0.077)	(0.065)
*Reciprocity*									
Reciprocity	0.0092**	0.0138***	0.0119***	0.0199***	0.0210***	0.0078	0.1710***	0.1553***	0.0600**
	(0.004)	(0.004)	(0.004)	(0.008)	(0.008)	(0.006)	(0.029)	(0.029)	(0.025)
*Handwashing/Hygiene Practices in April*									
Average of four/fifteen items			0.0966***			0.6580***			0.5600***
			(0.007)			(0.012)			(0.013)
Observations	4,501	4,501	4,501	4,501	4501	4501	4501	4501	4501

In this table, handwashing practices measured in April (1st Survey) and July/August (4th Survey) are first used separately as the outcome variables (the first and second column under each set of outcomes), and the handwashing practices measured in July/August are then used while those in April are additionally controlled for (the third column under each set of outcomes). Results yielded by Eq. (1) are presented in Panel A. Note that in Panel A, childhood handwashing experiences at shrines/temples and reciprocity (customs/reciprocity) are intentionally excluded from these findings because these two sets of variables are considered as concomitant variables which are a plausible surrogate for more immediately relevant confounding variables (education / residence). Individual characteristics, behavioral factors, labor-related variables, and age/prefecture dummies are also controlled for (for more detailed information, see table A3). Standard errors are given in parentheses. ***, **, and * indicate *p* < .01, *p* < .05, and *p* < .1

The results reported in Columns 1 and 4 of Table 3 relate to handwashing with water, and using soap, respectively, and are similar to those pertaining to the handwashing practices before the pandemic reported in Table 2. It is worth noting that, although education/residence and customs/reciprocity are all significantly associated with handwashing practices, the coefficients are smaller during the pandemic, indicating that more people had the sense of crisis during the state of emergency. These significant associations are also found with a wide range of hygiene practices, as shown in Column 7, suggesting education/residence and customs/reciprocity are associated with the overall hygiene practices beyond hand washing. While both concomitant and confounding variables are significant factors of either handwashing or hygiene practices, the size of the coefficients is larger in the results related to the latter. However, considering the fact that hygiene practices are measured on a 0 − 7 scale, the magnitudes of the coefficients of the aforementioned factors could be considered very similar between these two outcome variables.

For comparison, we then examined the same model using the fourth survey dataset. While more people were aware of the importance of hand hygiene practices at home and outside, some individuals might have already developed the so-called “COVID fatigue” as having to a wear mask in public places, while socially distancing and adhering to other health guidelines, was highly restrictive. The results of applying Eq. (1) and Eq. (2) to the fourth survey dataset (Columns 2, 5, and 8) indicate that the coefficients of confounding (education/residence) and concomitant (customs/reciprocity) variables remain significant, and the magnitudes of the coefficients are similar, suggesting the increasing the sense of crisis among the public overweighs the feeling of fatigue.

To examine the links of our main variables with long-lasting strict adherence to hand hygiene practices during the pandemic, particularly after the state of emergency was lifted, we estimated the hand hygiene practices in July/August after controlling for the values related to behaviors during April (Columns 3, 6, 9). Our results indicate that participants’ regular handwashing practices observed in April are the most significant factors of their subsequent hand hygiene compliance (captured in the survey conducted in July/August), implying that people who more strictly adhered to the hand hygiene regulations in April continued with self-compliance in July/August. Moreover, we find that some of the confounding and concomitant variables are also still significantly associated with both handwashing and hygiene practices, implying that the strict compliance with the hand hygiene policies even after the abolishment of the state of emergency is more likely among individuals that have had relevant childhood education and experiences, and have developed high levels of reciprocity.

Lastly, we briefly report here the results regarding individual and family characteristics to compare them with those found prior to the pandemic in Table 2 (see the results reported in Appendix 4). Demographic variables are similarly correlated with handwashing and hygiene practices during the pandemic. Comparatively, the volunteer activities and the low time discount rate that yielded mixed results in Table 2 in terms of the direction of the coefficients are only positively associated with the handwashing and hygiene practices during the pandemic, as expected. These findings support the significant association with the behavioral traits (e.g., higher altruism and lower time preference) as found in some previous studies (e.g., [9]).

## Discussion

### Implications of the links with education, culture, and reciprocity

Our analyses indicate that childhood education/experiences and reciprocity are significantly associated with current self-regulating hand hygiene attitudes in Japan. While no causal relationships can be derived from these findings, they may suggest that well-designed school education could allow students to exhibit more positive attitudes toward personal and communal hygiene during childhood and beyond. Thus, it is likely that such positive childhood handwashing education would be conductive to wearing a mask, preventing spreading of infection through touch, refraining from making clusters, and adopting various self-care measures during a pandemic. Furthermore, our results suggest that hygiene education could be more beneficial when it is used in conjunction with other intervention components, such as strengthening teachers’ engagement in hygiene-related initiatives both within and outside classroom, and developing interactive teaching methods that promote personal hygiene (e.g., handmade soap-making class, exercises involving dining table etiquette). We posit that these and similar school programs [38–40]^8^ would increase the likelihood that students would develop beneficial attitudes toward hand hygiene, and would gain greater appreciation of its role in personal and community health [12].

It is also worth noting that, although invisible and often difficult to identify, the prevailing cultural norms are significantly associated with personal attitudes toward daily hygiene. Therefore, analyzing cultural factors can be important for identifying how people in different regions formulate the attitudes toward hygiene at the individual level. Interestingly, our analyses suggest that even a limited exposure to handwashing experience at shrines is associated with greater adherence to handwashing practices (as established when comparing respondents that washed hands as a part of purification ritual only a few times relative to those who never did). Furthermore, as shown in Tables 2 and 3, participants that selected “often” and “always” when responding to the survey question probing into their childhood handwashing practices at shrines are more likely to wash their hands with soap as well as water. This significant link between the childhood cultural experiences at shrines and people’s everyday hand hygiene practices implies that exposure to the handwashing customs at shrines during childhood is not limited to the ritual experiences but may extend to more positive attitudes toward overall hygiene in daily life even among people who could potentially assign greater value to hand hygiene. These potential associations between the purification ritual customs and daily hygiene routines are also observed in other regions with different religious beliefs and norms (e.g., [20, 41, 42]).^9^

In contrast to the handwashing education at school and handwashing experiences at shrines that could be directly related to the likelihood that an individual would wash hands in all relevant situations, the role of childhood living environment in current hand hygiene practices may not be so obvious. Nonetheless, in our analyses, we posit that living in close proximity to shrines in childhood is likely to play a role in people’s attitudes toward reciprocal inclinations. As shown in Panel B of Table 2 (as well as in Appendix 4), reciprocity reduced the size of pertinent coefficients of living environment near shrines, implying that these factors are interrelated, as suggested by Ito et al. [25]. This association is also supported by our additional evidence suggesting a direct link between living near shrines as a child and reciprocal inclinations, which was statistically significant even after controlling for other demographic and labor-related variables (undocumented). Furthermore, our findings indicate that those who lived close to shrines in childhood visited shrines more frequently, as were thus more likely to partake in purification customs than their counterparts that did not live near shrines.^10^

Lastly, our analyses suggest that positive reciprocal inclinations could be associated with individuals’ disposition toward more stringent self-regulating behaviors at times of health crises, as this link remains significant even after adjusting for other related confounders (such as childhood education and experiences). In other words, individuals that tend to care for others would likely comply with public health policies even if adherence is not mandatory. Furthermore, as discussed above, as reciprocal inclinations can be developed through childhood education and experiences, these other-regarding preferences are also indirectly associated with hand hygiene attitudes through the confounders (i.e., education/residence), suggesting that, as hypothesized in the empirical framework, reciprocity as the concomitant variable lies on the direct path between confounding and outcome variables.

### Retrospective responses and recall bias

As questions related to childhood education and experiences required retrospective responses, our estimation results could suffer from the recall bias. For example, it is likely that those who pay more attention to personal hygiene would have a stronger impression of the hygiene education received in childhood, which would be reflected in their survey responses. However, several important attempts were made to mitigate such issues.

First, the survey questions probing into the childhood education/experiences were carefully worded to avoid obvious connection between the topics discussed and current handwashing practices. Specifically, the questions used to gather the data pertaining to the confounding variables (education/residence) probed into teachers’ involvement in students’ discipline, class content, and childhood living environment, rather than respondents’ actual handwashing behavior in childhood.^11^ In particular, we postulated that the childhood sociodemographic information (i.e., whether shrines/temples were in close proximity to the respondents’ home/route to school and were constantly in line of sight) can be recalled with a more sufficient degree of accuracy, and would less be linked to the current hygiene behavior than other retrospective variables.^12^ It is also worth noting that we provided “do not remember” as one of the multiple choices and confirmed that gender and age are not potentially associated with these retrospective responses (Appendix 6).^13^ Furthermore, we also assessed the robustness of our results by applying the models to subsamples comprising of young and old cohorts, confirming that the results are in general consistent with the main findings.^14^

Still, we acknowledge that the adoption of an indirect retrospective measure cannot completely eliminate the risk of recall bias. For example, some people might recall their childhood experiences *better* than others, which would be particularly problematic if those with a more vivid memory responded to the survey items pertaining to their childhood experiences more positively even though their childhood experiences did not differ from those of the respondents that had less accurate recollection of this period. This would mean instead that it would not cause a significant problem if those with a better memory responded more positively because they indeed had more experiences than their counterparts.

One possible scenario derived from incorrect recall is that some respondents who lived near shrines could have more vivid memories of their handwashing experiences at shrines, which could also be potentially biased by their current handwashing practices. Nonetheless, we argue that the possible recall bias of childhood handwashing experiences in relation to the childhood residence would not play a significant role in the estimation results, given that those who lived near shrines were more likely to *visit* shrines as children compared to those who did not live near shrines. This distinction suggests that better recollection of handwashing experiences at shrines should arise as a result of more frequent visits to shrines in childhood (and possibly, more exposure to purification rituals) relative to those who did not live near shrines. Consequently, even if our data is subject to recall bias, the information pertaining to childhood handwashing experiences (in relation to their residence) can be said to adequately capture the *total* amount of exposure to ritual customs (e.g., frequency of visits and/or handwashing experiences), and that is indeed what we aimed to measure.

Moreover, if responses to the survey questions related to childhood experiences were obtained based on inaccurate recall, similar effects would be observed in responses related to both shrines and temples. However, in most cases, the coefficients regarding shrines are only significantly correlated with Japanese people’s handwashing practices (Tables 2 and Appendix 4). We attribute these disparities to a more obvious role of Shinto shrines in the development of the spiritual and socio-psychological values of the neighborhood [43], which could create social and personal norms related to hand hygiene and care for others’ wellbeing. In contrast, while temples also feature prominently in the life of Japanese people, they tend to be regarded as spaces for meditative practices or rituals, such funerals, which would therefore promote stronger relationships with the ancestors and family members. Thus, if these differences in the role of shrines and temples are appropriately reflected in our estimation results, we postulate that respondents recalled their childhood residence quite accurately.

### Other confounders and social desirability bias

It is possible that our respondents developed the hygiene habits examined in this study at home, but our data unfortunately do not allow us to examine the *direct* link with parental hygiene attitudes and home discipline. While acknowledging that the family’s religious devotion (that requires purification rituals) is not a reflection of the aforementioned parental hygiene disciplines, we investigated its link with current handwashing practices by adding two dummy variables constructed from the responses to the following statements: (1) “There was a Shinto altar in my house when I was a child”; and (2) “There was a Buddhist altar in my house when I was a child.” A household Shinto altar (*kamidana* in Japanese) is a shelf where “apportioned spirits” (*bunrei*) of the Gods are enshrined, while Buddhist altars are tables with offerings to enshrine ancestral tablets (*ihai*) in household Buddhist altars (*butsudan*). As these altars are linked to handwashing practices, we posit that having altars in their childhood home might have (albeit partly) played a role in their family’s attitudes toward daily hand hygiene. When these dummy variables were included into Eq. (1) and (2), we found that most coefficients related to the education/residence and customs did not change significantly, even though their size was reduced in a few cases (undocumented). This finding suggests that religious devotion practices at home are not meaningfully related to handwashing education received in school and childhood experiences related to shrines.

Considering that regular handwashing is considered a desirable behavior, particularly at times of health crises, when such practices may be prescribed in government-issued guidelines, our survey respondents might have been reluctant to admit that their adherence to this practice is less than optimal, which would introduce social desirability bias into our results. Extant research has cautioned that the data derived from self-reports to *direct* survey questions about the compliance with social rules in place during the pandemic can be tainted by social desirability bias (e.g., [44, 45]), while several recent studies provide evidence suggesting that the estimates based on such self-report surveys do not significantly suffer from this problem [46–48]. To alleviate concerns over the reliability and validity of our results, in line with the work of Daoust et al. [45], suggesting the efficacy of using a question to soften the social norms of compliance, we probed into the practice of carrying a handkerchief, which was intentionally included in the survey as an additional (seemingly unrelated) question. As a handkerchief (or hand towel) is used in Japan on a regular basis for diverse purposes for wiping sweats, covering one’s mouth (or a part of face) when communicating with others, protecting one’s clothes (or table) when dining, as well as for drying hands after washing [49, 50], it might not be immediately associated solely with hand hygiene. Accordingly, although we associated propensity for carrying a handkerchief with high hygiene standards, this link would not be apparent to the survey participants. The results reported in Appendix 7 indicate that handwashing education at elementary school is also significantly correlated with this alternative indicator of handwashing practices, i.e., carrying a handkerchief outside home. In particular, those who always washed their hands at shrines tend to carry their handkerchief with them at all times, likely because only ladles for water were provided at the shrine entrance without any means of drying one’s hands and face.

Furthermore, to mitigate the effect of measurement errors that would result from not reading questions carefully, one of the survey items required participants to select the far-left choice (among 5 − 1 options, which should be 5). As those that failed to make a correct choice would likely read other questions with less care, we repeated our main estimations using the data provided by including a dummy variable which equals 1 for those that selected 5 (and 0 otherwise) and found these results comparable to the main estimation findings (undocumented).

Despite several important attempts to mitigate the potential biases derived from the survey design and data, our estimation results need to be interpreted with caution, and no causality should be assumed.

## Conclusions

Given that the data used in our analyses and the study design preclude any conclusions regarding the *causal* links that childhood education and experiences might have with hygiene practices, further research is needed to disambiguate this relationship. These concerns notwithstanding, we still believe that our findings could contribute to the better understanding of the role of these and other personal factors in the variations in *voluntary* compliance with strict hand hygiene practices. Thus, they can serve as a foundation for additional investigations aiming to assist with the formulation of the most optimal strategies in any future pandemics or other health crises.

## Supplementary Information

**Additional file 1: Appendix 1.** Mean Values of Handwashing Practices (0/1) with Water (left) and with Soap (right) from Survey 1 to 4. **Appendix 2.** Mean Values of Hygiene Practices (0−7 Scale) from Survey 1 to 4. **Appendix 3.** Descriptive Statistics of Independent Variables (n=6,050). **Appendix 4.** Handwashing Practices in April (Four Items Presented Separately). **Appendix 5.** Hygiene Practices in April (Fifteen Items Presented Separately). **Appendix 6.** Distribution of Handwashing Education and Childhood Residence by Age Group and Gender. **Appendix 7.** Usage of a Handkerchief.

## Data Availability

The datasets used and analyzed during the current study available from the corresponding author on reasonable request.

## Footnotes

1 It is noteworthy that in each wave, we displayed nudge messages related to contact avoidance and infection prevention to examine the nudge effects on subsequent behaviors as a part of a separate study. Clearly, as the nudge-based messages were provided in the first wave after the submission of responses regarding hand hygiene practices, and these nudge inventions targeted randomly chosen subjects, they should thus not impact our results in any of the subsequent waves. We nonetheless confirmed that there were no statistically significant differences between the individual characteristics (e.g., age, gender, education, income, etc.) and outcome variables of those who were exposed to the nudge messages and those who were not, and we controlled for each nudge message by introducing dummies into our models.

2 As the government has not mandated any strict self-restraint regulations for certain regions or cities during the survey periods, no significant regional differences in terms of hand hygiene practices were expected. We nonetheless estimated the model with additional adjustments of current residence (and also confirmed that the regional differences do *not* significantly change the results).

3 To further elucidate the role of education and handwashing practices, we examined four situations that could differ slightly in their implications for personal and communal hygiene. For example, handwashing before meals is less of a social act compared to washing hands after toilet use.

4 For household income, as some participants selected “do not know” or “do not want to respond” from the provided list of responses, we constructed household income as dummies to account for these cases. It is worth noting that those that were not employed at the time of the survey would select “others” when responding to work-related questions and hence, non-responses did not exist in our dataset.

5 To be more specific, when examining the link between respondents’ hygiene attitudes and elementary school education received by their children in comparison to those with children at other levels of school education, we segregated the sample as follows: children aged 0 − 5 (9.93%), children aged 6 − 12 attending elementary school (5.55%), children aged 12 − 18 (5.44%), aged ≥ 18 (26.30%), and without children (52.78%).

6 As we postulate that four situations for handwashing could differ slightly in their implications for personal and communal hygiene, the negative correlation between reciprocal inclinations and washing before meals before the pandemic can be interpreted as indicating handwashing before meals is less considered as a social act compared to other situations, at least before the pandemic.

7 In addition to those who have children attending elementary school, those with children attending nursery/preschool and secondary school are more likely to wash hands with *soap* than those with adult children (aged 18 +) or childless individuals, possibly due to the greater emphasis on early childhood and school education in the family attitudes at home (similar results can be found in the table of Appendix 4).

8 For example, training teachers to remind elementary students to wash their hands at specified times during the school day, clearly marked paths to handwashing stations, and visual prompts, such as posters depicting areas of the hands to scrub have been shown to promote good hand hygiene among elementary school students.

9 Most of the cited studies emphasize that, even if religious norms are a significant factor in individual attitudes toward hygiene, these are more strongly associated with easy access to water supply and sanitation services, hygiene promotion in media, and development of locally embedded hygiene programs/campaigns, particularly for the disadvantaged sections of population in the given countries.

10 While approximately 98.1% of those who lived close to shrines in childhood indicated that they had visited shrines during this period, a lower percentage (approximately 91%) of their counterparts that did not live near shrines as children had this experience while growing up.

11 Moreover, these questions were presented at the end of the survey after a long series of demographic questions. As they were separated from the questions probing into the respondents’ current hand hygiene behavior, we postulated that respondents would be less likely to link their answers to the items related to childhood handwashing practices to those pertaining to their current behaviors.

12 The rationale behind this view was that, as shrines/temples are the places for important life events of Japanese individuals (e.g., weddings, funerals, etc.), handwashing should not feature prominently in their recollections of those events when prompted by questions about their childhood living environment near shrines/temples. Furthermore, it is noteworthy that according to our data, 49% (36.4%) of the respondents indicated that they lived near shrines (temples), suggesting that such living environments are rather common in Japan.

13 Neither gender nor age generally related to the propensity for selecting the “do not remember” response, with the exception of some possible treatment imbalance. As an attempt to resolve this potential issue, “Do not remember” dummy was added to our regressions, along with various controls.

14 We applied the models to subsamples comprising of individuals aged ≤ 44 (the mean age of our sample) and 44 + years, as the former group would be more likely to recall their childhood experiences accurately. Based on our undocumented findings, the relationships examined in our work persist in both groups, but are nonetheless stronger for the younger cohort.
